# Adherence to a Dietary Approaches to Stop Hypertension (DASH)-type diet over the life course and associated vascular function: a study based on the MRC 1946 British birth cohort

**DOI:** 10.1017/S0007114517003877

**Published:** 2018-03-14

**Authors:** Jane Maddock, Nida Ziauddeen, Gina L. Ambrosini, Andrew Wong, Rebecca Hardy, Sumantra Ray

**Affiliations:** 1 MRC Unit for Lifelong Health & Ageing at UCL, 33 Bedford Place, London WC1B 5JU, UK; 2 MRC Elsie Widdowson Laboratory, Cambridge CB1 9NL, UK; 3 Academic Unit of Primary Care and Population Sciences, Faculty of Medicine, University of Southampton, South Academic Block, Southampton General Hospital, Tremona Road, Southampton SO16 6YD, UK; 4 NNEdPro Global Centre for Nutrition and Health (affiliated with: Cambridge University Health Partners and the British Dietetic Association), St John’s Innovation Centre, Cowley Road, Cambridge CB4 0WS, UK; 5 School of Population and Global Health, The University of Western Australia, 35 Stirling Highway, Crawley, Perth, WA 6009, Australia

**Keywords:** Dietary Approaches to Stop Hypertension diet, Vascular function, British birth cohort, Life course epidemiology

## Abstract

Little is known about long-term associations between the Dietary Approaches to Stop Hypertension (DASH) diet and conventional cardiovascular (CV)-risk factors as well as novel measures of vascular function. This study aimed to examine whether long-term adherence to a DASH-type diet in a British birth cohort is associated with conventional CV-risk factors and two vascular function markers, carotid intima–media thickness (cIMT) and pulse wave velocity (PWV). Data came from 1409 participants of the Medical Research Council (MRC) National Survey of Health and Development. Dietary intake was assessed at 36, 43, 53 and 60–64 years using 5-d estimated food diaries. The DASH-type diet score was calculated using the Fung index. Conventional CV-risk factors (blood pressure (BP) and lipids), cIMT in the right and/or left common carotid artery and PWV was measured when participants were 60–64 years. Associations between the DASH-type diet score and outcomes were assessed using multiple regression models adjusted for socioeconomic position, BMI, smoking and physical activity. Participants in higher sex-specific quintiles (Q) of the long-term DASH-type diet had lower BP (*P*≤0·08), higher HDL-cholesterol (*P*<0·001) and lower TAG (*P*<0·001) compared with people in Q1. Participants in Q5 of the long-term DASH-type diet had lower PWV (−0·28 sd; 95 % CI −0·50, −0·07, *P*_trend_=0·01) and cIMT (−0·24 sd; 95 % CI −0·44, −0·04, *P*_trend_=0·02) compared with participants in the Q1. This association was independent of the conventional CV-risk factors. Greater adherence to a DASH diet over the life course is associated with conventional CV-risk factors and independently associated with cIMT and PWV.

CVD remains a significant public health problem worldwide despite reductions in mortality in high-income countries^(^ ^1^ ^)^. Many modifiable and non-modifiable factors have been implicated in the aetiology of CVD. Diet is one such modifiable factor. The association between diet and CVD has been studied extensively, but evidence that supports the role of specific foods or nutrients remains equivocal. This lack of consistent evidence is partly due to the complexities of measuring diet as an exposure. An individual’s diet consists of a variety of foods which contain an assortment of nutrients and non-nutrients that can act interactively, synergistically or cumulatively to affect the initiation and/or progression of a disease^(^ ^2^ ^)^. Examining the effect of overall diet on CVD instead of single nutrients may capture the collective benefits of this heterogeneous exposure.

A number of different diets, foods and nutrients have been identified as being cardio-protective, including the Dietary Approaches to Stop Hypertension (DASH) diet^(^ ^3^ ^)^. The DASH diet is characterised by high proportions of fruit, vegetables, low-fat dairy products and whole-grain foods and is low in saturated fat and refined sugar^(^ ^4^ ^)^. The DASH diet was designed for an 8-week trial to compare the effect of consuming the average US diet *v*. a diet high in fruit and vegetables or the DASH diet, on blood pressure (BP)^(^ ^4^ ^)^. Both diets lowered BP compared with the average US diet, with people adhering to the DASH diet experiencing the greatest BP reduction^(^ ^4^ ^)^. Observational studies have since shown that adherence to a DASH-type diet is associated with a lower incidence of stroke, heart failure and heart disease^(^ ^5^ ^,^ ^6^ ^)^.

The most compelling evidence to date for the cardio-protective mechanism of DASH is through its BP-lowering effect. In a meta-analysis of seventeen randomised controlled trials (RCT; *n* 2561, duration 2–26 weeks), the DASH diet reduced systolic BP (SBP) by 6·74 mmHg (95 % CI −8·25, −5·23 ) and diastolic BP (DBP) by 3·54 mmHg (95 % CI, −4·29, −2·79). The greatest effect was seen among hypertensive participants and in studies where the interventions were designed to restrict energy intake^(^ ^7^ ^)^. Limiting Na intake increases this BP-lowering effect of DASH^(^ ^8^ ^)^. A number of meta-analyses of RCT have been conducted which examine the role of DASH in relation to other CVD risk factors. Soltani *et al*.^(^ ^9^ ^)^ (ten studies, *n* 1291, duration=8–24 weeks) showed that adults on a DASH diet lost 1·42 kg (95 % CI −2·03, −0·28) more weight on average compared with controls. Authors of another meta-analysis (twenty studies, *n* 1917, duration=2–24 weeks) observed a decrease in total cholesterol and LDL-cholesterol, but not glucose, HDL-cholesterol or TAG. Changes were greatest among those with higher baseline BP or BMI^(^ ^10^ ^)^. Given that the majority of evidence for the cardio-protective role of the DASH diet comes from trials, its long-term effect on conventional cardiovascular (CV)-risk factors, such as, BP and lipids remains unknown.

The role of the DASH diet in novel aetiological CVD pathways is also under-explored. Vascular and endothelial dysfunction represent an integrated CVD risk pathway^(^ ^11^ ^)^. Dysfunction of the vascular endothelium is characterised by damage to its barrier function, impaired vasodilator responses, thrombogenesis and increased expression of adhesion molecules with leucocytosis. This dysfunction is considered a key initial event in atherogenesis^(^ ^12^ ^)^. Carotid intima–media thickness (cIMT), a non-invasive ultrasound biomarker of early atherosclerosis^(^ ^13^ ^)^ and carotid-femoral pulse wave velocity (PWV), a measure of arterial compliance as well as stiffness^(^ ^14^ ^)^ are two complementary measures of vascular function that have been used to predict CVD risk in the general population. There is some limited evidence mainly from RCT that the DASH diet and/or its components can affect these markers of vascular function^(^ ^15^ ^–^ ^18^ ^)^. Examining the association between adherence to a DASH-type diet and these novel vascular function markers may provide useful insights into the role of diet in the early stages of CVD and reveal opportunities for early CVD prevention.

This study aims to use data from a British birth cohort to examine whether long-term adherence to a DASH-type diet is associated with: (1) conventional CV-risk factors; (2) two markers of vascular function: cIMT and PWV.

## Methods

### Participants

**Fig. 1:**
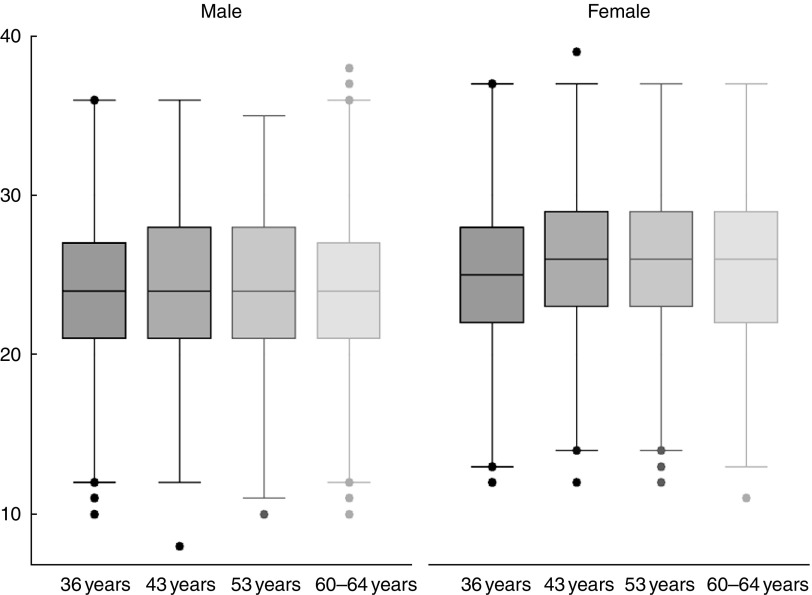
Dietary Approaches to Stop Hypertension-type diet score per year by sex of the 769 participants with dietary data from all time points.

We used data from participants of the Medical Research Council (MRC) National Survey of Health and Development (NSHD). The original NSHD sample consisted of 5362 babies born in 1 week in March 1946 to married parents in England, Scotland or Wales, stratified by social class^(^ ^19^ ^)^. This sample has been followed up twenty-four times since birth^(^ ^20^ ^,^ ^21^ ^)^. At the 23rd follow-up, eligible study members (*n* 2856) were invited to complete a clinical assessment at one of six clinical research facilities or home visitation by a nurse when they were 60–64 years. Invitations were not sent to those who had died (*n* 778), who were living abroad (*n* 570), had previously withdrawn from the study (*n* 594), or had been lost to follow-up (*n* 564). A total of 2229 participants responded of whom 76 % (*n* 1690) attended a clinic and 24 % (*n* 539) had a home visit. Although this sample was broadly representative of the national population at a similar age, individuals attending the clinical assessment had lower adiposity and lifetime smoking exposure and higher levels of physical activity compared with the original cohort^(^ ^22^ ^)^. Information about participants’ smoking habits at 69 years was used to impute missing values at 60–64 years as described below. For the main analyses in this study we included participants who had information for at least one of the vascular measures at 60–64 years (1565 for cIMT and 1257 for PWV) and who had dietary information from at least two adult time points (1335 for cIMT and 1081 for PWV). A flow chart of participants included in our study can be found in the online Supplementary Fig. S1. Ethical approval was obtained from the Greater Manchester and the Scotland Research Ethics Committees and participants provided written and informed consent (Fig. 1).

### Dietary Approaches to Stop Hypertension-type diet

Information about dietary intake was collected using 5-d estimated diet diaries when participants were aged 36, 43, 53 and 60–64 years^(^ ^23^ ^–^ ^27^ ^)^. All food and drink consumed were coded in MRC Human Nutrition Research Cambridge using the in-house programmes, Diet In Data Out and Diet In Nutrients Out. Nutrient intakes were calculated based on McCance and Widdowson’s The Composition of Foods^(28)^. A total of 2411, 2256, 1772 and 1869 participants at 36, 43, 53 and 60–64 years, respectively, completed ≥3 d of the diet diaries. When generating the DASH-type diet in NSHD, we included all participants with dietary information regardless of whether they attended the clinical assessment at 60–64 years.

We calculated the DASH score in accordance with previously published methods by Fung *et al*.^(^ ^5^ ^)^. The DASH score was based on the original studies of the DASH diet focusing on eight components in particular: high intake of fruits (including pure fruit juice), vegetables (excluding potatoes), nuts and legumes, low-fat dairy products, wholegrains and low intake of red and processed meats, Na and sweetened beverages (including sugar sweetened cordial and sweetened fruit based drinks)^(^ ^5^ ^)^. These components consisted of discrete foods as well as disaggregated foods^(^ ^29^ ^)^. Details of what was included in each of these components can be found in the online Supplementary Table S1. Each individual’s DASH score was calculated for each year using the following steps: (1) the energy density for each component was calculated, for example fruit (g)/energy (4184 kJ (1000 kcal))^(^ ^30^ ^)^; (2) each component was classified into quintiles (Q), where fruits, vegetables, nuts and legumes, low-fat dairy products and wholegrains were assigned 1–5 points in order of most consumption. Quintiles for red and processed meats, free sugar and Na were assigned 1–5 points in order of least consumption; (3) the quintiles for each component were summed to obtain an overall DASH score which could range from 8 to 40; (4). As the DASH score was higher in women (mean difference=2·3; 95 % CI 1·9, 2·8), the overall DASH score was grouped into sex-specific quintiles (Q1–Q5) for use in regression models.

We calculated a cumulative average of all DASH scores over the 24–28-year period for each individual who had dietary information from at least two time points (*n* 2477). This represents long-term adherence to the DASH-type diet^(^ ^31^ ^)^. This mean, long-term DASH score was also grouped into sex-specific quintiles (Q1–Q5).

### Cardiovascular risk factors

SBP and DBP were measured twice using a HEM-705 sphygmomanometer (Omron) at the 60–64 years clinic visits. The second reading (or first if missing) was used for analysis. Total cholesterol, HDL, TAG and LDL were also measured in fasting blood samples as previously described^(^ ^20^ ^,^ ^32^ ^)^. Information on the use of antihypertensive and lipid-lowering drugs was self-reported at 60–64 years.

### Vascular measures at 60–64 years

Vascular measures were obtained by trained research staff during the clinical assessment only when participants were 60–64 years. PWV was measured using the Vicorder (Skidmore Medical) by placing a 10-cm wide pressure cuff at the upper right thigh and 3 cm partial cuff over the right carotid artery. Cuffs were inflated to 65 mmHg for 10–15 s. Path length was measured between the cuffs and defined as the distance between the suprasternal notch directly to the top of the femoral cuff. PWV was automatically calculated by an integral algorithm. cIMT was measured in the left and right common carotid artery using a high-resolution imaging scanner (Vivid I, GE Healthcare (12 MHz probe)) with a 12 MHz probe^(^ ^33^ ^,^ ^34^ ^)^. Participants with carotid plaque, cIMT >1·5 mm with abnormal shape and wall texture, were excluded (*n* 13)^(^ ^34^ ^)^. A combined average of left and right cIMT is used as one outcome. To increase comparability, each outcome was standardised to a mean of 0 and sd of 1.

### Covariates

Socioeconomic position (SEP) at age 53 years (or 43 years if missing) was grouped into: professional and intermediate; skilled non-manual; semi-skilled and unskilled manual.

BMI was calculated using the standard formula with information from height and weight measured at 60–64 years. Physical activity in adulthood was self-reported. Participants were categorised as inactive (reported no participation); moderately active (participated in relevant activity one to four times/month) or most active (participated in relevant activities five or more times/month) using information at 36, 43, 53 and 60–64 years. The overall physical activity score was the sum of responses at each time point, ranging from 0 to 8^(^ ^35^ ^)^. Smoking status at 60–64 years was categorised as never, current or ex-smoker at 60–64 years (or 53 or 69 years if missing (*n* 115)).

### Statistical analysis

First, a series of regression models determined if long-term adherence to a DASH-type diet (modelled as sex-specific quintiles) associated with: antihypertensive medication, lipid-lowering medication, DBP, SBP, total cholesterol, LDL-cholesterol, HDL-cholesterol, and TAG at 60–64 years. We used logistic regression models when antihypertensive or lipid-lowering medications were the outcomes and linear regression when HDL was the outcome. We used censored normal regression when SBP, DBP, TAG, total cholesterol or LDL-cholesterol were the outcomes. Individuals taking antihypertensive or lipid-lowering medication were censored at their observed value. This assumes that the underlying BP or lipid levels in an individual taking medication is at least as high as their observed value and aims to reduce bias and a loss of statistical power^(^ ^36^ ^)^. TAG were not normally distributed so log transformed values were used in the model. All models were adjusted for SEP, BMI, smoking and physical activity. Log likelihood ratio tests assessed deviation from linearity by comparing models with the DASH quintiles fitted as a categorical variable to models with DASH quintiles fitted as a continuous variable.

Second, regression models assessed associations between long-term adherence to a DASH-type diet and the standardised vascular function outcomes: cIMT and PWV. Models were adjusted for SEP, BMI, smoking and physical activity. Deviation from linearity was tested using log likelihood ratio tests. Interactions between the DASH-type diet and sex on the vascular outcomes were also assessed. Models were re-run separately for individual DASH scores at 36, 43, 53 and 60–64 years, adjusting for the covariates mentioned above. In order to maximise power, these analyses did not exclude participants with dietary information available from only one time point.

Third, models that assessed long-term adherence to a DASH-type diet and vascular function were additionally adjusted for CV-risk factors found to be associated with the DASH-type diet in step one. This step assessed the potential for a mediating role of CV-risk factors on the relationship between the DASH-type diet and vascular function.

All analyses were performed using STATA version 14.

## Results

A total of 1409 participants (52 % women) had at least one measure of cIMT or PWV at 60–64 years and had dietary information from at least two time points between 36 and 60–64 years (online Supplementary Fig. S1). These participants were more physically active, smoked less and had a lower BMI compared with people attending the clinical assessment who did not have this dietary and vascular measure information (*n* 820).

Mean cIMT and PWV were slightly higher for men compared with women, whereas DASH scores were higher for women (Table 1). Mean DASH scores did not change noticeably over time (Table 1). Figure one illustrates DASH scores from each year for the 769 participants who had dietary data at all time points. The online Supplementary Table S2 summarises distributions of each DASH component according to quintiles of the sex-specific DASH quintiles.

**Table 1:** Descriptives of participants with at least one vascular measure and dietary information from more than one time point (*n* 1409)(Numbers and percentages; mean values and standard deviations)

	Men (*n* 673)				Women (*n* 736)			
	*n* *	%	Mean	sd	*n* *	%	Mean	sd
Socioeconomic status at 53 years (or 43 years if missing)								
Professional and managerial	429	64·3			323	45·2		
Skilled non-manual	67	10·0			260	36·4		
Skilled manual	124	18·6			43	6·1		
Semi-skilled and unskilled	47	7·1			89	12·5		
BMI at 60–64 years (kg/m^2^)	673		27·6	3·8	736		27·3	5·0
Smoking status at 60–64 years (or 53/69 years if missing)	543		10·8	15·3	585		7·7	13·2
Current	61	9·2			63	8·6		
Ex-smoker	409	61·5			394	53·3		
Never smoker	195	29·3			279	38·1		
Self-reported physical activity since 1982								
Inactive at all ages	71	10·6			96	13·0		
Active at least once/month	551	81·9			605	82·2		
Active more than once/month	51	7·6			35	4·8		
Antihypertensive medication at 60–64 years								
No	463	71·0			547	75·6		
Yes	189	29·0			177	24·5		
Lipid-lowering medication at 60–64 years								
No	501	74·4			623	84·7		
Yes	172	25·6			113	15·4		
Vascular phenotype at 60–64 years								
Systolic blood pressure (mmHg)	672		138·8	18·1	732		132·1	7·4
Diastolic blood pressure (mmHg)	673		78·9	10·0	732		75·5	9·4
Total cholesterol (mmol/l)	648		5·3	1·1	697		6·1	1·2
LDL-cholesterol (mmol/l)	627		3·3	0·9	684		3·8	1·0
HDL-cholesterol (mmol/l)	648		1·4	0·3	697		1·8	0·4
TAG (mmol/l)	632		1·4	0·9	686		1·1	0·6
Carotid intima–media thickness (mm)	639		0·71	0·15	696		0·67	0·11
Pulse wave velocity (m/s)	501		8·31	1·41	580		8·03	1·57
DASH-type diet score								
Long-term DASH score †	673		23·5	3·9	736		25·8	3·9
Mean DASH score at 36 years	554		23·4	4·7	615		25·0	5·1
Mean DASH score at 43 years	572		23·8	4·8	628		26·2	4·8
Mean DASH score at 53 years	478		23·5	5·1	575		26·4	4·9
Mean DASH score at 60–64 years	632		23·7	5·1	700		25·9	5·1

DASH, Dietary Approaches to Stop Hypertension.

* Missing values not presented.

† Average from 36, 43, 53 and 60–64 years in individuals with ≥2 d of dietary information.

### Long-term Dietary Approaches to Stop Hypertension score and cardiovascular risk factors

Associations between long-term DASH scores and CVD risk factors was as expected, that is those in the higher quintiles of the long-term DASH-type diet had a favourable CVD risk factor profile compared with those in the lowest quintile. However, there was no evidence of an association for total or LDL-cholesterol (Table 2). Adjustment for SEP, BMI, smoking and physical activity attenuated associations, but evidence for a relationship between a long-term DASH-type diet and SBP, lipid-lowering medication, HDL-cholesterol and TAG remained (Table 2). There was also a tendency for lower DBP with increasing DASH quintiles remaining following adjustment for covariates (*P*_trend_=0·08).

**Table 2:** Association between long-term Dietary Approaches to Stop Hypertension (DASH) scores (sex-specific quintiles) and classic cardiovascular risk factors* unadjusted (Coefficients and 95 % confidence intervals)

			DASH Q2		DASH Q3		DASH Q4		DASH Q5		
	*n*	DASH Q1	Coefficient	95 % CI	Coefficient	95 % CI	Coefficient	95 % CI	Coefficient	95 % CI	*P*_trend_ †
Unadjusted											
Antihypertensive medication‡	1841	Ref.	0·97	0·65, 1·28	0·68	0·36, 1·00	0·68	0·36, 1·00	0·56	0·23, 0·89	<0·001
Diastolic blood pressure§	1825	Ref.	−0·35	−2·21, 1·51	−1·13	−2·93, 0·67	−1·78	−3·57, 0·02	−3·09	−4·89, −1·30	<0·001
Systolic blood pressure§	1824	Ref.	−1·98	−5·40, 1·45	−4·60	−7·93, −1·28	−5·39	−8·70, −2·08	−7·70	−11·00, −4·40	<0·001
Lipid-lowering medication‡	1883	Ref.	0·93	0·60, 1·25	0·62	0·28, 0·96	0·63	0·29, 0·97	0·45	0·08, 0·81	<0·001
Total cholesterol§	1746	Ref.	−0·07	−0·25, 0·12	−0·02	−0·20, 0·15	−0·09	−0·26, 0·09	−0·15	−0·32, 0·03	0·10
LDL-cholesterol§	1670	Ref.	−0·08	−0·24, 0·08	−0·02	−0·18, 0·13	−0·06	−0·21, 0·10	−0·07	−0·22, 0·08	0·50
HDL-cholesterol	1746	Ref.	0·05	−0·01, 0·12	0·10	0·04, 0·17	0·11	0·04, 0·17	0·15	0·08, 0·21	<0·001
ln TAG§	1679	Ref.	−7·27	−16·56, 2·02	−24·51	−33·50, −15·52	−25·73	−34·77, −16·69	−41·35	−50·30, −32·40	<0·001
Adjusted for SEP, BMI, smoking and physical activity											
Antihypertensive medication‡	1774	Ref.	0·98	0·64, 1·32	0·73	0·39, 1·08	0·82	0·48, 1·17	0·78	0·41, 1·15	0·12
Diastolic blood pressure§	1759	Ref.	−0·35	−2·19, 1·50	−0·60	−2·41, 1·21	−0·97	−2·80, 0·86	−1·59	−3·49, 0·31	0·08
Systolic blood pressure§	1758	Ref.	−1·93	−5·35, 1·49	−3·45	−6·81, −0·08	−3·62	−7·01, −0·23	−4·83	−8·35, −1·31	0·01
Lipid-lowering medication‡	1813	Ref.	1·01	0·66, 1·36	0·68	0·32, 1·05	0·76	0·39, 1·12	0·61	0·21, 1·02	0·01
Total cholesterol§	1687	Ref.	−0·13	−0·32, 0·06	−0·07	−0·25, 0·12	−0·11	−0·30, 0·08	−0·16	−0·35, 0·04	0·19
LDL-cholesterol§	1617	Ref.	−0·13	−0·30, 0·04	−0·05	−0·22, 0·11	−0·07	−0·23, 0·10	−0·06	−0·23, 0·11	0·83
HDL-cholesterol	1687	Ref.	0·03	−0·03, 0·09	0·05	−0·01, 0·11	0·04	−0·03, 0·10	0·05	−0·02, 0·11	<0·001
ln TAG§	1626	Ref.	−2·89	−11·88, 6·09	−13·84	−22·64, −5·04	−13·35	−22·31, −4·39	−22·59	−31·85, −13·33	<0·001

Q, sex-specific quintile; Ref., referent values.

* *N*’s not restricted to those with carotid intima–media thickness or pulse wave velocity measures.

† Linear trend test, that is DASH quintiles fitted as continuous exposure in regression model. No evidence for deviation from linear tend using log likelihood ratio test, that is testing DASH quintiles fitted as continuous exposure *v*. DASH quintiles fitted as categorical exposure, *P*≥0·17 for all models.

‡ Logistic regression, OR presented.

§ Censored regression to account for medication use.

### Dietary Approaches to Stop Hypertension score and vascular function at 60–64 years

Participants in higher quintiles of long-term DASH-type diet had better vascular function than those in the lowest quintile after adjustment for SEP (Table 3). The association remained statistically significant following adjustment for BMI, smoking and physical activity. There was no evidence for an interaction between long-term DASH score and sex (*P≥*0·32) for either cIMT or PWV. There was no evidence for deviation from a linear trend between the long-term DASH score and PWV and cIMT (*P*≥0·50).

**Table 3:** Long-term Dietary Approaches to Stop Hypertension (DASH) score and vascular function (Coefficients and 95 % confidence intervals)

	Standardised carotid intima–media thickness*		Standardised pulse wave velocity†	
	Coefficient	95 % CI	Coefficient	95 % CI
DASH score sex-specific quintiles (Q)				
Model 1||				
Q1	Ref	Ref	Ref	Ref
Q2	−0·08	−0·28, 0·12	−0·13	−0·35, 0·09
Q3	−0·15	−0·34, 0·04	−0·07	−0·28, 0·14
Q4	−0·18	0·37, 0·01	−0·18	−0·39, 0·02
Q5	−0·35	−0·54, −0·16	−0·30	−0·51, −0·10
	*P*_trend_ ‡ =<0·001		*P*_trend_ ‡=0·003	
	*P*_deviation from trend_ §=0·72		*P*_deviation from trend_ §=0·52	
Model 2||				
Q1	Ref.	Ref.	Ref.	Ref.
Q2	−0·06	−0·26, 0·14	−0·13	−0·35, 0·09
Q3	−0·10	−0·29, 0·09	−0·06	−0·27, 0·15
Q4	−0·10	−0·29, 0·09	−0·17	−0·38, 0·04
Q5	−0·24	−0·44, −0·04	−0·28	−0·50, −0·07
	*P*_trend_ ‡ =0·02		*P*_trend_ ‡ =0·01	
	*P*_deviation from trend_ §=0·74		*P*_deviation from trend_ §=0·50	

Ref., referent values.

* cIMT model 1: *n* 1309 model 2: *n* 1298.

† PWV model 1: *n* 1061 model 2: *n* 1051.

‡ Linear trend test, that is DASH quintiles fitted as continuous exposure in regression model.

§ Log likelihood ratio test, that is testing DASH quintiles fitted as continuous exposure *v*. DASH quintiles fitted as categorical exposure.

|| Model 1 adjusted for socioeconomic position; model 2 additionally adjusted for BMI, smoking and physical activity.

Prospective and cross-sectional associations between DASH scores at each follow-up and PWV were in the same direction but generally weaker compared with results for long-term DASH scores (online Supplementary Table S3). The strongest prospective association between DASH scores and cIMT was at 36 years. This association was bigger in magnitude compared with long-term DASH scores (online Supplementary Table S3).

### Long-term Dietary Approaches to Stop Hypertension score, vascular function and cardiovascular risk factors at 60–64 years

There was no evidence that CV-risk factors which showed an association with DASH-type diet in this study (i.e. DBP, SBP, lipid-lowering medication, HDL-cholesterol and TAG) explain the association between long-term DASH scores and vascular function (Table 4).

**Table 4:** Long-term Dietary Approaches to Stop Hypertension (DASH) score and vascular function adjusted for conventional cardiovascular risk factors|| (Coefficients and 95 % confidence intervals)

	Standardised carotid intima–media thickness*		Standardised pulse wave velocity†	
DASH score sex-specific quintiles (Q)	Coefficient	95 % CI	Coefficient	95 % CI
Q1	Ref.	Ref.	Ref.	Ref.
Q2	−0·04	−0·24, 0·16	−0·14	−0·35, 0·08
Q3	−0·08	−0·27, 0·11	−0·07	−0·27, 0·13
Q4	−0·07	−0·26, 0·13	−0·13	−0·33, 0·07
Q5	−0·24	−0·44, −0·04	−0·24	−0·45, −0·04
	*P*_trend_ ‡=0·02		*P*_trend_ ‡=0·03	
	*P*_deviation from trend_ §=0·52		*P*_deviation from trend_ §=0·54	

* cIMT: *n* 1220.

† PWV: *n* 980.

‡ Linear trend test, that is DASH quintiles fitted as continuous exposure in regression model.

§ Log likelihood ratio test, that is testing DASH quintiles fitted as continuous exposure *v*. DASH quintiles fitted as categorical exposure.

|| Adjusted for socioeconomic position, BMI, smoking and physical activity, diastolic blood pressure, systolic blood pressure, lipid-lowering medication, HDL-cholesterol, TAG.

## Discussion

We showed that long-term adherence to a DASH-type diet is associated with a favourable CV-risk profile in adulthood using data from a British birth cohort. We also observed a relationship between long-term adherence to a DASH-type diet and improved vascular function, which was not explained by the conventional CV-risk factors.

There is substantial evidence from RCT for the role of the DASH diet in reducing BP^(^ ^4^ ^,^ ^7^ ^,^ ^8^ ^)^ and our study supports this. However, the magnitude of the estimated relationship between long-term adherence to DASH and BP in this longitudinal observational study is smaller than estimates from RCT^(^ ^7^ ^)^. In this study, we observed SBP to be 4·83 mmHg (95 % CI −8·35, −1·31) and DBP to be 1·59 mmHg (95 % CI 0 −3·49, 0·31) lower among people in the highest DASH-type diet quintile compared with the lowest quintile. Results from a meta-analysis of RCT showed an average reduction which was 6·74 mmHg (95 % CI −5·23, −8·25) in SBP and 3·54 mmHg (95 % CI −4·29, −2·79) in DBP greater among people consuming a DASH diet compared with controls^(^ ^7^ ^)^. This difference in effect size is likely due to the differences in length of exposure, specification of the DASH diet and study design. To our knowledge, we were the first to demonstrate an association between a DASH-type diet and other conventional CV-risk factors (i.e. HDL-cholesterol, TAG) in a longitudinal observational study. This finding was not fully supported by a meta-analysis of RCT^(^ ^10^ ^)^. Differences in study design, that is RCT lasting 2–24 weeks *v*. longitudinal observational study assessing a 24–28-year period, is the likely cause of this inconsistency.

Our findings are also the first to demonstrate that greater adherence to a DASH-type diet over a 24–28-year period in adulthood is associated with a greater vascular function as measured by the vascular function markers, PWV and cIMT. There are a limited number of studies investigating the association between a DASH-type diet or its key dietary components and PWV. These studies are largely supportive of our results. Findings from an intervention study demonstrated a reduction in arterial stiffness among nineteen normotensive participants consuming a low-Na DASH diet compared with the consumption of a diet low in fruit and vegetables over a 3-week period^(^ ^15^ ^)^. Authors from another RCT including 144 overweight and obese participants (forty-nine participants consuming a DASH diet and enrolled in a weight management programme; forty-six consuming standard DASH diet; forty-nine on a control diet), found that people in the two DASH diet interventions had lower PWV compared with controls^(^ ^16^ ^)^. To our knowledge, there is no previous observational evidence for the association between a DASH diet and PWV. A review has highlighted that a number of DASH components, including high fruit and vegetable and dairy product composition have been associated with lower PWV providing further support for our findings^(^ ^17^ ^)^. Compared with PWV, we observed a slightly weaker association between the DASH-type diet and cIMT, an early marker of atherosclerosis. In 2014 Petersen *et al*.^(^ ^18^ ^)^ conducted a review of the association between cIMT and individual dietary components and dietary patterns . Although they found observational evidence for an association between higher fruit and whole-grain consumption, lower saturated fat consumption (all components of a DASH-type diet) and lower cIMT, they emphasised that the evidence is currently weak^(^ ^18^ ^)^.

PWV has been shown to predict CVD events beyond conventional risk factors such as those measured by the Framingham risk score, including BP^(^ ^37^ ^–^ ^39^ ^)^. Although a single measure of cIMT has been shown to predict CVD, it has been shown to have no predictive value beyond conventional CV-risk factors^(^ ^13^ ^,^ ^40^ ^)^. We observed that the relationship between the DASH-type diet and both measures of vascular function was not explained by conventional CV-risk factors (SBP, DBP, lipid-lowering medication, HDL-cholesterol and TAG) and therefore associations are not likely to be acting through these mechanisms. There are novel mechanisms, for example inflammation and oxidative stress that have also been implicated in the relationship between a DASH-type diet and CVD^(^ ^5^ ^,^ ^41^ ^–^ ^43^ ^)^ which may explain the relationship between the DASH-type diet and vascular function.

The longitudinal design of NSHD allowed us to carry out secondary analysis to examine the potential for sensitive periods in adulthood in which the consumption of a DASH-type diet might be most beneficial for vascular function. The association between the DASH diet and PWV did not differ greatly over time. However, the association between greater adherence to a DASH diet at 36 and 43 years and cIMT was stronger than any other time point. This suggests that consuming a DASH-type diet from earlier-adulthood may be particularly important for cIMT.

The major strengths of this study were the use of a nationally representative birth cohort of the white British population born in the early post World War II period^(^ ^19^ ^,^ ^22^ ^)^ and detailed repeated measures of dietary intake as assessed by diet diaries over a 24–28-year period. Our sample for this study had a slightly healthier lifestyle (i.e. smoked less, exercised more and had a lower BMI) compared with the remaining study participants. As this was an observational study, exact replication of the original DASH diet^(^ ^4^ ^)^ was not possible. However, we used the Fung DASH index^(^ ^5^ ^)^ which has been shown to perform equally well as other indices^(^ ^44^ ^)^. Although we did not observe major changes in adherence to the DASH-type diet over time, a previous study demonstrated that participants of NSHD have healthier diets the older they become^(^ ^26^ ^)^. Cautious interpretation of study findings are required since calculation of the DASH-type diet in this study is relative to this specific study sample. For example, if the majority of the study sample did not eat in accordance with the DASH diet, even those in the highest quintile would have low adherence to the DASH diet, which may have weakened observed associations. Miller and colleagues published standards for maximum scores for defining a DASH-type diet using different DASH indices, including the Fung index^(^ ^44^ ^)^. The participants in the highest DASH quintile in our study were not quite meeting these standards^(^ ^44^ ^)^. For example, participants in the top quintile in our study consumed approximately 3 servings of fruit/d, 2·6 servings of vegetables/d (one portion is equivalent to 80 g) and 3 servings of wholegrains/d (one portion is equivalent to 16 g) per 8368 kJ (2000 kcal). The maximum standard servings/d based on 8368 kJ (2000 kcal) as outlined by Miller are;≥4 servings of fruit/d, ≥3 vegetables/d and ≥4 servings of wholegrains/d.

In conclusion, we found observational evidence for an association between adherence to a DASH-type diet over an approximately 30-year period and better conventional CV-risk factors. We also demonstrated an association between long-term adherence to a DASH-type diet and two vascular function markers, cIMT and PWV, at 60–64 years which was not explained by the conventional risk factors. This study provides further evidence for the beneficial role of a DASH-type diet in CVD risk reduction; however replication in other longitudinal observational cohorts is required.

## Supplementary material

For supplementary material accompanying this paper visit http://dx.doi.org/10.1017/S0007114517003877. click here to view supplementary material
