# Supplementary material for: Adherence to a Dietary Approaches to Stop Hypertension (DASH)-type diet over the life course and associated vascular function: a study based on the MRC 1946 British birth cohort
Source: Br J Nutr. 2018 Mar 14;119(5):581–9. doi: 10.1017/S0007114517003877 (PMC5848753; doi:10.1017/S0007114517003877)
Supplement: Supplementary file 1 [file S0007114517003877sup001.docx]

**Adherence to a DASH-type diet over the life course and vascular function in the MRC 1946 British birth cohort: Supplementary Material**


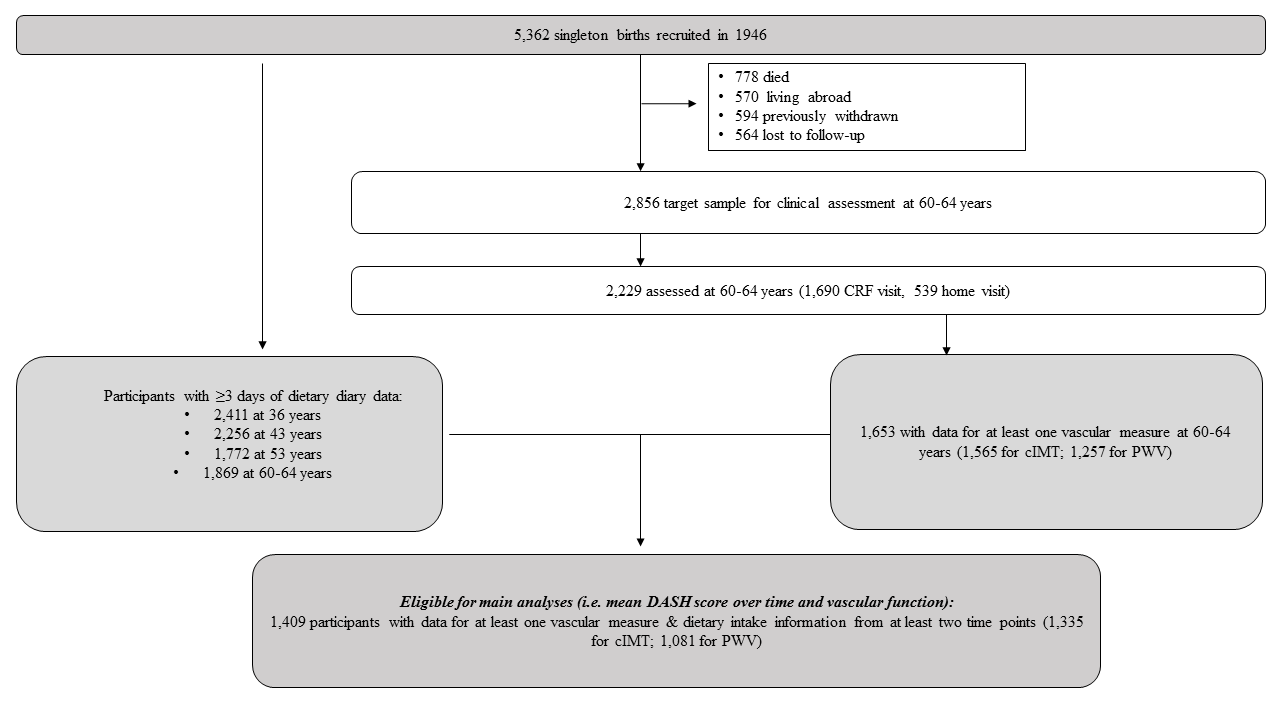


**Supplementary figure 1.** Number of participants in the National Survey of Health and Development and selection for the present study. CRF: clinical research ~~resrach~~ facility; cIMT: carotid intima-media thickness; PWV: pulse wave velocity

| **Supplementary table 1**: Components of each DASH food group based on Fung et al. (1) | |
| --- | --- |
| **DASH food group** | **Food group component (grams)** |
| Fruit | Fruit* (including fresh, canned and cooked) + dried fruit* + pure fruit juices^†^ |
| Vegetables | Tomatoes + tomato puree + brassicaceae + yellow, red or green vegetables + other vegetables^‡^ |
| Nuts and legumes | Beans + nuts |
| Low-fat dairy^§^ | Reduced or low fat yoghurts and drinking yoghurts + low fat, skimmed, semi-skimmed milk |
| Whole grains | Wholegrain includes foods in unprocessed state that still has the endosperm, bran and germ |
| Red and processed meat ^\|^ | Beef + lamb + pork + processed red meat + all other red meat + burgers + sausages + processed poultry |
| Sweetened beverages | Carbonated soft drinks + sugar sweetened fruit drinks + squash/concentrated fruit drink |
| Sodium | Sodium naturally occurring in foods and added to ready meals and purchased products. Salt added at the table or during cooking is also included if quantified by the participant e.g. one teaspoon salt in a recipe and had one-fourth of the recipe but not if stated as salt to taste in the recipe. |
| * Includes disaggregated fruit components of desserts, pastries etc.  ^†^ excluding sugar sweetened fruit drinks, e.g. sugar sweetened cordial ~~squash~~/concentrated fruit drink  ^‡^ excluding potatoes  ^§^ Excludes reduced fat ice cream and dairy desserts  ^\|^ Includes disaggregated red and processed meat components of composite dishes. Processed red meat included bacon and ham | |

| **Supplementary table 2**: Components of long-term DASH (i.e. average DASH scores for an individual from all available follow ups over 24-28 years, *n*=2,447)* | | | | | | | | | | | | | | | | |
| --- | --- | --- | --- | --- | --- | --- | --- | --- | --- | --- | --- | --- | --- | --- | --- | --- |
|  | Fruit, | | Vegetables, g/1,000kcal | | Nuts & legumes g/1,000kcal | | Low-fat dairy g/1,000kcal | | Wholegrain g/1,000kcal | | Red/processed meat g/1,000kcal | | Sweetened beverages g/1,000kcal | | Sodium mg/1,000kcal | |
|  | g/1,000kcal | |  |  |  |  |  |  |  |  |  |  |  |  |  |  |
|  | Mean | (SD) | Mean | (SD) | Mean | (SD) | Mean | (SD) | Mean | (SD) | Mean | (SD) | Mean | (SD) | Mean | (SD) |
| DASH score (range) ^†^ | |  |  |  |  |  |  |  |  |  |  |  |  |  |  |  |
| Q1 (12-22) | 33.6~~56~~ | (27.5~~49~~) | 62.7~~68~~ | (27.1~~1~~) | 3.81 | (3.94) | 47.0~~1~~ | (48.5~~48~~) | 5.43 | (6.04) | 50.5~~49~~ | (16.1~~2~~) | 59.7~~66~~ | (63.9~~3~~) | 1412~~.10~~ | (245~~.25~~) |
| Q2 (19-24) | 55.3~~28~~ | (401.0~~99~~) | 74.2~~1~~ | (30.1~~08~~) | 4.85 | (4.64) | 66.8~~0~~ | (52.5~~48~~) | 9.22 | (7.37) | 42.2~~3~~ | (13.8~~2~~) | 42.5~~3~~ | (51.5~~49~~) | 1357~~.10~~ | (211~~0.62~~) |
| Q3 (21-26) | 77.9~~1~~ | (49.7~~66~~) | 79.3~~3~~ | (31.5~~48~~) | 5.61 | (7.22) | 75.1~~4~~ | (47.0~~6.99~~) | 13.61 | (8.23) | 38.3~~25~~ | (13.6~~58~~) | 34.8~~0~~ | (44.9~~4~~) | 1332~~1.91~~ | (188~~7.84~~) |
| Q4 (24-28) | 89.9~~89~~ | (49.9~~0~~) | 89.5~~45~~ | (37.0~~6.95~~) | 6.60 | (5.39) | 92.1~~1~~ | (57.0~~6.99~~) | 17.51 | (10.29) | 33.8~~3~~ | (12.7~~66~~) | 28.1~~07~~ | (33.5~~45~~) | 1302~~.29~~ | (181~~0.97~~) |
| Q5 (26-35) | 119.9~~2~~ | (53.6~~59~~) | 105.9~~87~~ | (43.7~~3~~) | 8.67 | (6.89) | 107.9~~0~~ | (58.1~~0~~) | 24.52 | (10.75) | 25.1~~09~~ | (13.0~~1~~) | 21.0~~3~~ | (30.3~~28~~) | 1278~~.00~~ | (190~~89.63~~) |
| ^*^ Including all participants with dietary information regardless of whether they attended the clinical assessment at 60-64 years | | | | | | | | | | | | | | | | |
| ^†^ range overlaps due to sex-specific groupings | | | | | | | | | | | | | | | | |

| **Supplementary table 3A**: DASH scores at 36y and vascular function at 60-64y | | | | | | | | |
| --- | --- | --- | --- | --- | --- | --- | --- | --- |
|  |  | **Standardised carotid intima media thickness*** | | |  | **Standardised pulse wave velocity†** | | |
|  |  | Coef | (95% CI) | |  | Coef | (95% CI) | |
| **DASH score sex-specific fifths (Q)** | | |  |  |  |  |  |  |
| *Model 1* | Q1 | Ref | Ref | |  | Ref | Ref | |
|  | Q2 | -0.15 | (-0.36, | 0.05) |  | 0.15 | (-0.07, | 0.38) |
|  | Q3 | -0.26 | (-0.45, | -0.06) |  | 0.02 | (-0.19, | 0.23) |
|  | Q4 | -0.30 | (-0.51, | -0.09) |  | -0.08 | (-0.30, | 0.15) |
|  | Q5 | -0.33 | (-0.52, | -0.13) |  | -0.07 | (-0.28, | 0.15) |
|  |  | p_trend_^‡^ = <0.001 | | |  | p_trend_ ^‡^ =0.10 | | |
|  |  |  |  |  |  |  |  |  |
| *Model 2* | Q1 | Ref | Ref | |  | Ref | Ref | |
|  | Q2 | -0.14 | (-0.35, | 0.06) |  | 0.17 | (-0.06, | 0.40) |
|  | Q3 | -0.20 | (-0.39, | -0.003) |  | 0.04 | (-0.18, | 0.26) |
|  | Q4 | -0.24 | (-0.45, | -0.03) |  | -0.06 | (-0.29, | 0.17) |
|  | Q5 | -0.27 | (-0.47, | -0.07) |  | -0.06 | (-0.28, | 0.16) |
|  |  | p_trend_ ^‡^ =0.01 | | |  | p_trend_ ^‡^ =0.12 | | |
|  |  | p deviation from trend^§^ = 0.81 | | |  | p deviation from trend^§^ =0.29 | | |
| Model 1 adjusted for socioeconomic position; Model 2 additionally adjusted for BMI, smoking and physical activity  *cIMT model 1: *n*=1,111 model 2: *n*=1,102  ^†^ PWV model 1: *n*=900 model 2: *n*=892  ^‡^ Linear trend test i.e. DASH quintiles fitted as continuous exposure in regression model  ^§^ Log likelihood ratio test i.e. testing DASH quintiles fitted as continuous exposure vs. DASH quintiles fitted as categorical exposure | | | | | | | | |

| **Supplementary table 3B**: DASH scores at 43y and vascular function at 60-64y | | | | | | | | |
| --- | --- | --- | --- | --- | --- | --- | --- | --- |
|  |  |  | | |  |  | | |
|  |  | **Standardised carotid intima media thickness*** | | |  | **Standardised pulse wave velocity^†^** | | |
|  |  | Coef | (95% CI) | |  | Coef | (95% CI) | |
| **DASH score sex-specific fifths (Q)** | | |  |  |  |  |  |  |
| *Model 1* | Q1 | Ref | Ref | |  | Ref | Ref | |
|  | Q2 | -0.19 | (-0.39, | 0.001) |  | -0.06 | (-0.27, | 0.15) |
|  | Q3 | -0.30 | (-0.49, | -0.10) |  | -0.06 | (-0.27, | 0.15) |
|  | Q4 | -0.20 | (-0.39, | -0.01) |  | -0.16 | (-0.36, | 0.04) |
|  | Q5 | -0.30 | (-0.50, | -0.11) |  | -0.22 | (-0.43, | -0.01) |
|  |  | ptrend ‡=0.01 | | |  | ptrend ‡=0.02 | | |
|  |  |  |  |  |  |  |  |  |
| *Model 2* | Q1 | Ref | Ref | |  | Ref | Ref | |
|  | Q2 | -0.14 | (-0.34, | 0.05) |  | -0.04 | (-0.25, | 0.17) |
|  | Q3 | -0.25 | (-0.45, | -0.05) |  | -0.04 | (-0.25, | 0.18) |
|  | Q4 | -0.13 | (-0.32, | 0.06) |  | -0.15 | (-0.36, | 0.05) |
|  | Q5 | -0.21 | (-0.41, | -0.01) |  | -0.19 | (-0.41, | 0.03) |
|  |  | p_trend_ ^‡^=0.10 | | |  | p_trend_ ^‡^=0.04 | | |
|  |  | *p* deviation from trend^§^ = 0.23 | | |  | *p* deviation from trend^§^ =0.90 | | |
| Model 1 adjusted for socioeconomic position; Model 2 additionally adjusted for BMI, smoking and physical activity  *cIMT model 1: *n*=1,131 model 2: *n*=1,122  ^†^ PWV model 1: *n*=920 1 model 2: *n*=912  ^‡^ Linear trend test i.e. DASH quintiles fitted as continuous exposure in regression model  ^§^ Log likelihood ratio test i.e. testing DASH quintiles fitted as continuous exposure vs. DASH quintiles fitted as categorical exposure | | | | | | | | |

| **Supplementary table 3C**: DASH scores at 53y and vascular function at 60-64y | | | | | | | | |
| --- | --- | --- | --- | --- | --- | --- | --- | --- |
|  |  |  | | |  |  | | |
|  |  | **Standardised carotid intima media thickness*** | | |  | **Standardised pulse wave velocity^†^** | | |
|  |  | Coef | (95% CI) | |  | Coef | (95% CI) | |
| **DASH score sex-specific fifths (Q)** | | |  |  |  |  |  |  |
| *Model 1* | Q1 | Ref | Ref | |  | Ref | Ref | |
|  | Q2 | -0.09 | (-0.30, | 0.12) |  | -0.04 | (-0.27, | 0.20) |
|  | Q3 | -0.08 | (-0.30, | 0.13) |  | -0.25 | (-0.49, | -0.01) |
|  | Q4 | -0.24 | (-0.45, | -0.03) |  | -0.24 | (-0.46, | -0.01) |
|  | Q5 | -0.14 | (-0.35, | 0.08) |  | -0.27 | (-0.50, | -0.03) |
|  |  | p_trend_ ^‡^=0.07 | | |  | p_trend_ ^‡^=0.01 | | |
|  |  |  |  |  |  |  |  |  |
| *Model 2* | Q1 | Ref | Ref | |  | Ref | Ref | |
|  | Q2 | -0.03 | (-0.25, | 0.18) |  | -0.02 | (-0.25, | 0.21) |
|  | Q3 | -0.01 | (-0.22, | 0.21) |  | -0.22 | (-0.46, | 0.01) |
|  | Q4 | -0.15 | (-0.36, | 0.06) |  | -0.19 | (-0.42, | 0.04) |
|  | Q5 | -0.04 | (-0.26, | 0.17) |  | -0.22 | (-0.45, | 0.02) |
|  |  | p_trend_ ^‡^ =0.37 | | |  | p_trend_ ^‡^=0.02 | | |
|  |  | *p* deviation from trend^§^ = 0.49 | | |  | *p* deviation from trend^§^ =0.57 | | |
| Model 1 adjusted for socioeconomic position; Model 2 additionally adjusted for BMI, smoking and physical activity  *cIMT model 1: *n*=991 model 2: *n*=991  ^†^ PWV model 1: *n*=812 model 2: *n*=812  ^‡^ Linear trend test i.e. DASH quintiles fitted as continuous exposure in regression model  ^§^ Log likelihood ratio test i.e. testing DASH quintiles fitted as continuous exposure vs. DASH quintiles fitted as categorical exposure | | | | | | | | |

| **Supplementary table 3D**: DASH scores at 60-64y and vascular function at 60-64y | | | | | | | | |
| --- | --- | --- | --- | --- | --- | --- | --- | --- |
|  |  | **Standardised carotid intima media thickness*** | | |  | **Standardised pulse wave velocity^†^** | | |
|  |  | Coef | (95% CI) | |  | Coef | (95% CI) | |
| **DASH score sex-specific fifths (Q)** | | |  |  |  |  |  |  |
| *Model 1* | Q1 | Ref | Ref | |  | Ref | Ref | |
|  | Q2 | -0.13 | (-0.31, | 0.04) |  | -0.10 | (-0.30, | 0.09) |
|  | Q3 | -0.26 | (-0.43, | -0.09) |  | -0.18 | (-0.36, | 0.01) |
|  | Q4 | -0.15 | (-0.33, | 0.02) |  | -0.16 | (-0.35, | 0.03) |
|  | Q5 | -0.24 | (-0.41, | -0.07) |  | -0.34 | (-0.53, | -0.16) |
|  |  | p_trend_ ^‡^ =0.01 | | |  | p_trend_ ^‡^ = <0.001 | | |
|  |  |  |  |  |  |  |  |  |
| *Model 2* | Q1 | Ref | Ref | |  | Ref | Ref | |
|  | Q2 | -0.09 | (-0.26, | 0.08) |  | -0.08 | (-0.27, | 0.11) |
|  | Q3 | -0.19 | (-0.36, | -0.02) |  | -0.13 | (-0.32, | 0.06) |
|  | Q4 | -0.05 | (-0.23, | 0.13) |  | -0.11 | (-0.31, | 0.08) |
|  | Q5 | -0.14 | (-0.32, | 0.04) |  | -0.27 | (-0.47, | -0.08) |
|  |  | p_trend_ ^‡^ =0.24 | | |  | p_trend_ ^‡^ =0.01 | | |
|  |  | *p* deviation from trend^§^= 0.19 | | |  | *p* deviation from trend^§^ =0.75 | | |
| Model 1 adjusted for socioeconomic position; Model 2 additionally adjusted for BMI, smoking and physical activity  *cIMT model 1: *n*=1,319 model 2: *n*=1,308  ^†^ PWV model 1: *n*=1,067 model 2: *n*=1,058  ^‡^ Linear trend test i.e. DASH quintiles fitted as continuous exposure in regression model  ^§^ Log likelihood ratio test i.e. testing DASH quintiles fitted as continuous exposure vs. DASH quintiles fitted as categorical exposure | | | | | | | | |

1. Fung TT, Chiuve SE, McCullough ML, Rexrode KM, Logroscino G, Hu FB. Adherence to a DASH-style diet and risk of coronary heart disease and stroke in women. Arch Intern Med. 2008;168(7):713-20.
